# The sphenoidal emissary foramina prevalence: a meta-analysis of 6,369 subjects

**DOI:** 10.1007/s00276-022-03051-1

**Published:** 2022-12-06

**Authors:** Maria Piagkou, Michael Kostares, Fabrice Duparc, Panagiotis Papanagiotou, Constantinus Politis, George Tsakotos, Nikos Pantazis, Konstantinos Natsis

**Affiliations:** 1 Department of Anatomy, School of Health Sciences, Department of Medicine, National and Kapodistrian University of Athens, 75 Mikras Asias Street, Athens, GR 11527 Greece; 2 Laboratory of Anatomy, Faculty of Medicine-Pharmacy, Rouen-Normandy University, Rouen, France; 3 First Department of Radiology, School of Health Sciences, National and Kapodistrian University of Athens, Aretaieion Hospital, Athens, Greece; 4 Department of Imaging and Pathology, Faculty of Medicine, KU Leuven, University Hospitals Leuven, 49 Herestraat, 3000 Louvain, Belgium; 5 Department of Hygiene, Epidemiology and Medical Statistics, School of Health Sciences, Department of Medicine, National and Kapodistrian University of Athens, Athens, Greece; 6 Department of Anatomy and Surgical Anatomy, Faculty of Health Sciences, School of Medicine, Aristotle University of Thessaloniki, Thessaloniki, Greece

**Keywords:** Foramen Vesalius, Sphenoidal emissary foramen, Prevalence, Meta-analysis, Laterality-specific prevalence

## Abstract

**Purpose:**

To estimate the prevalence of the sphenoidal emissary foramina (SEF), and the effect of possible moderators on it.

**Methods:**

A systematic online literature search was conducted. The pooled prevalence with 95% confidence intervals was estimated. Outlier and influential analyses were performed. The presence of small-study effect and publication bias were evaluated. Moderator analyses were executed to investigate the effect of the specimens’ continent of origin, type of study (dried skull or imaging), probing for the evaluation of SEF patency (conduction and instruments used), side dominance (bilateral or unilateral), morphometric data [SEF diameter, distances SEF–Foramen ovale (FO) and SEF–Foramen spinosum (FS)], and the methodology used for the morphometric measurements (caliper, DICOM Viewer, and image analysis software) on the estimated prevalence.

**Results:**

In total, 6,460 subjects from 26 studies were included in the meta-analysis. The overall SEF prevalence was estimated as 38.1%. The heterogeneity was high and statistically significant. No indications of publication bias and small-study effect were identified. The conducted subgroup analyses did not yield statistically significant differences in the SEF prevalence between groups, except of the type of side dominance. Both results of the univariable and multivariable regression analyses showed the association of the unilateral dominance with a decrease in the reported SEF prevalence.

**Conclusion:**

The identification of more unilateral than bilateral foramina in a given cohort is associated with a decrease in the reported crude SEF prevalence. Laterality-specific estimates should be established for a precisive estimation of the emissary foramina prevalence.

## Introduction

The constant foramina (foramina ovale and spinosum, FO and FS) are located in the posterior part of the greater wings of the sphenoid bone. FO transmits the mandibular nerve and occasionally the accessory meningeal artery and the lesser superficial petrosal nerve. FS is perforated by the middle meningeal artery and the meningeal branch of the trigeminal nerve [12]. Occasionally, anteromedially or anteriorly to the FO [19, 31], a small sized foramen—the so-called sphenoidal emissary foramen (SEF) or foramen of Vesalius (FV) can be unilaterally or bilaterally identified. SEF has not been identified in any other primates than human [40]. As per its content, SEF transmits a sphenoidal emissary vein (SEV) connecting the pterygoid venous plexus to the cavernous sinus [24]. Therefore, it is important in neurosurgical procedures, such as in FO cannulation for trigeminal nerve rhizotomy, as well as pathway of spreading of extracranial infections into the cavernous sinus [22, 25, 35]. The SEF occurrence varies widely among different studies’ samples [13].

The current meta-analysis provides a more precise estimation of the SEF prevalence and pinpoints the variables associated with the SEF presence.

## Materials and methods

### Search strategy

**Fig. 1:**
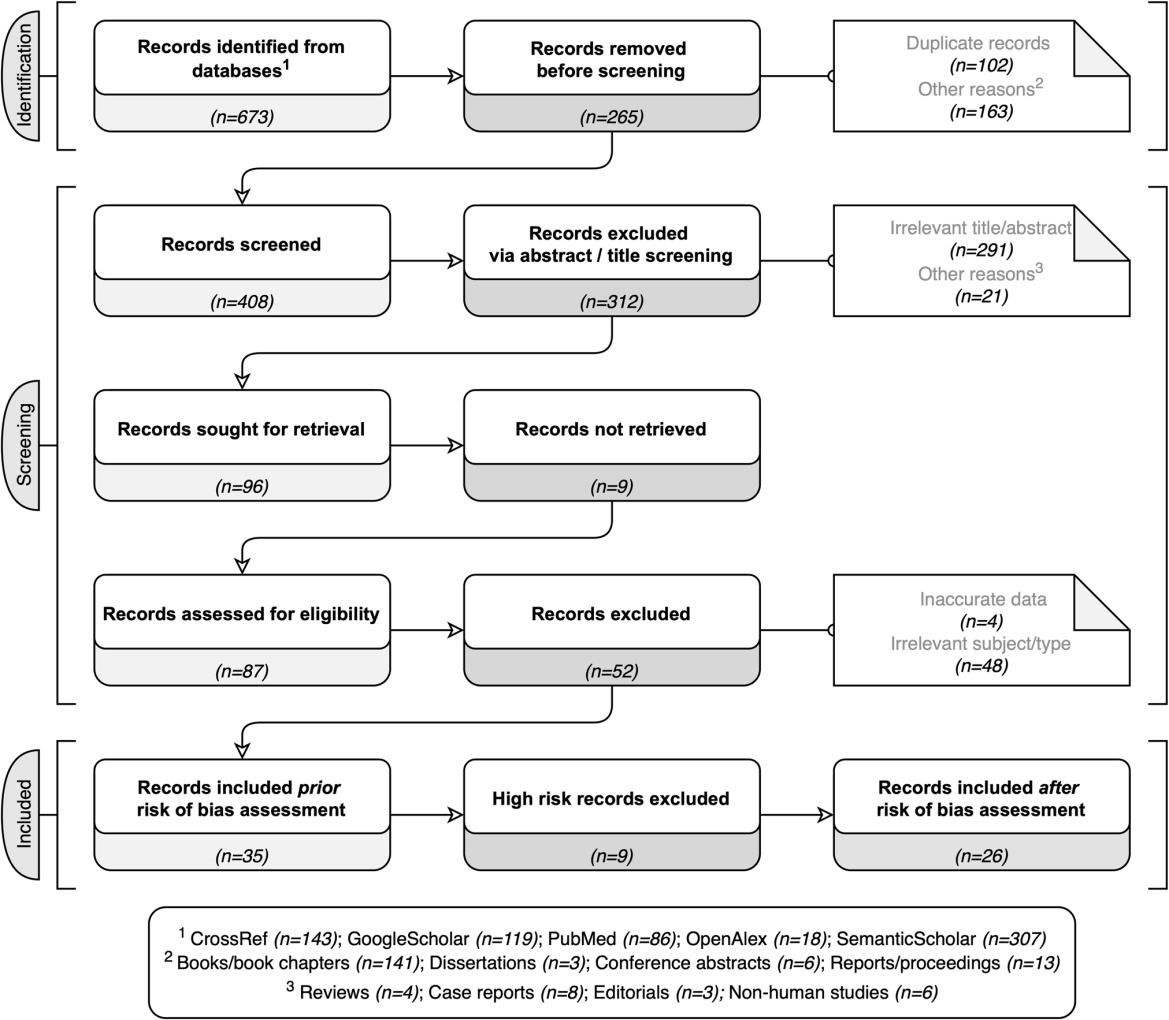
Flow chart depicting the systematic search results from the relevant studies' identification and selection

A systematic literature search was conducted by two independent assessors in August 2022 using Publish or Perish software [15]. Through this application, all available databases except for the *Web of Science* (*Crossref, GoogleScholar, OpenAlex, PubMed, Scopus, and Semantic Scholar*) were scanned using combinations of the following keywords: *[“foramen Vesalius”, “sphenoidal emissary foramen”, “presence”, “occurrence”, “prevalence”, “incidence”)].* Notably, in *Semantic Scholar* and *OpenAlex*, only single keywords were used since both databases’ application programming interfaces did not support the use of Boolean operators. After duplicates’ removal, each publication’s reference list was manually scanned for potentially non-identified studies. The systematic literature search flowchart (Fig. 1) is based on the PRISMA 2020 Statement [29].

### Criteria for study selection and data inclusion and extraction

All original studies reporting data regarding SEF prevalence were included with no restriction on language or publication date. Case reports, review articles, letters to the editor, conference abstracts, doctoral thesis, studies with no full-text or detailed abstracts available, and articles that could not be cross-verified by multiple secondary sources were excluded. Out of each publication, the extracted data included the *authors, year of publication, continent of origin (Europe, Asia, and America), type of data (dried skulls and imaging), probing for evaluating SEF patency (yes or no), instrument used for probing (bristle, wire, and other), total sample, reported SEF frequency (total, bilateral, and unilateral), type of dominance (bilateral: when the bilateral to unilateral foramina ratio was greater than 1, otherwise, unilateral), morphometric data (SEF diameter, SEF–FO, and SEF–FS distances),* and the *methodology used for the morphometric measurements (caliper, DICOM Viewer, image analysis software).* In publications not mentioning the continent of origin, the country where the study originated from, was eventually recorded and in case of an article written in a non-Latin language (e.g., Russian), the full paper was downloaded and translated using the Google Translate website (https://translate.google.com). Additionally, in manuscripts where only the bilateral or unilateral percentages were reported, the respective frequencies (bilateral and unilateral frequency) were calculated by converting each percentage to integers with no decimal approximation.

### Quality assessment

The quality assessment was performed according to the Anatomical Quality Assessment (AQUA) tool [16], a tool consisting of 25 questions and dividing into 5 areas: *1. Objectives and Subject Characteristics, 2. Study Design, 3. Methodology Description, 4. Descriptive Anatomy, and 5. Results Reporting.* For each domain, where all questions were replied affirmatively, the risk of bias was rated as 'low', otherwise as 'high'. Study quality was defined as ‘high’ if at low risk of bias in all five domains, ‘moderate’ if at low risk of bias at least in three domains, and otherwise as ‘low’.

### Statistical analysis

Statistical analysis was carried out using RStudio (version: 2022.7.1.554) software (RStudio Team (2022)). RStudio: Integrated Development for R. RStudio, PBC, Boston, MA for MacOS. The DerSimonian and Laird random-effects model was used to estimate the pooled prevalence and its respective 95% confidence intervals (CI). No logit or double arcsine transformation were made since the observed proportions identified across studies were between 0.2 and 0.8 [21, 37]. Heterogeneity presence across studies was estimated by constructing a forest plot and tested using the Cochran’s Q statistic and its respective *p* value. The Higgins *I*^2^ statistic and its respective 95% CI were used for quantifying the magnitude of true heterogeneity in effect sizes. An *I*^2^ value of 25%, 50%, and 75% indicated low, moderate, and high heterogeneity. To detect studies that overly contributed to the heterogeneity, a Baujat plot [2] was created. To determine if the potential outlying studies, as evaluated in this plot, were also influential, screening for externally studentized residuals with z-values larger than two in absolute value and leave-one-out diagnostics were performed [38]. With the outlying and influential studies removed, the pooled prevalence, its’ respective 95% CI, and the substantial heterogeneity were re-evaluated through moderator analyses. In the conducted subgroup analyses, the following covariates were evaluated: *continent of origin, type of data, probing, sample size, dominance, study quality,* and *measurements.* As per the *sample size*, all manuscripts were divided into two categories (small and large studies) based on the median sample size (*n* = 239 subjects). In the performed univariable regression analyses, except of the aforementioned covariates, the SEF diameter, as well as the SEF–FO and SEF–FS distances were assessed as per their relationship with the respective effect sizes. Moreover, the presence of interrelated moderators was checked to avoid potential multicollinearity issues prior the conduction of the multivariate regression analysis. Due to the limited availability of data about the SEF diameter, and the SEF-FO, and SEF-FS distances in the given dataset, they were not used in this analysis. To detect the presence of publication bias, a Doi plot and a funnel plot were created. The asymmetry of each plot was estimated by calculating the LFK index [9] and Egger’s tests’ *p* value, respectively. Additionally, to detect the presence of the small-study effect, the phenomenon that smaller studies may show different, often larger effects than large ones [33], a funnel plot of the prevalence against the sample size was constructed and regression-based Egger’s test was estimated. The arithmetic difference between percentages was expressed in percentage point units [39]. Unless otherwise stipulated, the statistical significance was established at *p* = 0.05 (two-tailed).

## Results and discussion

### Search results and characteristics of the included studies

A total of 26 studies (*n* = 6,460 subjects); 23 dried skull (*n* = 5,590 subjects) and 4 imaging (*n* = 870 subjects) were included. Thirteen studies (48.2%) were conducted in Asia, nine studies (36.3%) in America, and five (18.5%) in Europe. The majority of articles evaluated the SEF patency (15 studies, 55.6%). Out of them, seven studies (46.6%) reported the use of wires, three (20.0%) the use of bristles, and five (33.4%) the use of other materials, such as metallic probes or endodontic files. The 66.7% of the included studies were estimated as moderate quality and the remaining ones as high quality. The 51.9% of the studies that referred to the SEF had calculated its anteroposterior diameter by utilizing the use of calipers (six studies, 42.9%), DICOM viewers (three studies, 21.4%) or image analysis software (five studies, 35.7%). A list of the included studies is presented in Table 1.

**Table 1:** Main characteristics and data outcome of the included studies

Authors	Year	Continent	Study type	Probing	Total sample	SEF frequency			Dominance	Study size	Study Quality	Conduction of measurements	SEF morphometry (in mm)		
						T	B/L	U/L					SEF diameter	SEF–FO	SEF–FS
Alves and Deana [1]	2017	America	Dried skulls	No	178	57	43	14	Bilateral	Small	Moderate	Yes (caliper)		2.19	
Bayrak et al. [2]	2018	Asia	Imaging	No	317	89	22	67	Unilateral	Large	Moderate	Yes (DV)	2.74	2.26	11.29
Boyd [3]	1930	Europe	Dried skulls	No	1500	548	221	327	Unilateral	Small	Moderate	No			
Berlis et al. [4]	1992	Europe	Dried skulls	No	60	22	9	13	Unilateral	Large	Moderate	Yes (DV)			
Chaisuksunt et al. [5]	2012	Asia	Dried skulls	Yes (wire)	377	61	16	45	Unilateral	Large	High	Yes (IAS)	1.59	2.42	
Costa do Nascimento et al. [6]	2018	America	Dried skulls	No	194	36	12	24	Unilateral	Small	Moderate	Yes (caliper)			
Dogan et al. [7]	2014	Asia	Dried skulls	No	31	15	5	10	Unilateral	Small	Moderate	Yes (caliper)	2.46	3.61	
Ginsberg et al. [9]	1994	America	Imaging	No	123	98	60	38	Bilateral	Small	Moderate	No			
Görürgöz and Paksoy [10]	2020	Asia	Imaging	Yes (wire)	260	190	110	80	Bilateral	Large	High	Yes (DV)	1.75	1.39	10.32
Gupta et al. [12]	2005	Asia	Dried skulls	Yes (bristle)	35	15	8	7	Bilateral	Small	Moderate	No			
Gupta et al. [13]	2014	Asia	Dried skulls	Yes (bristle)	200	68	28	40	Unilateral	Small	Moderate	No			
Jadhav et al. [15]	2016	Asia	Dried skulls	Yes (bristle)	250	72	44	28	Bilateral	Large	High	No			
Kale et al. [16]	2009	Asia	Dried skulls	Yes (other)	347	156	87	69	Bilateral	Large	High	No			
Leonel et al. [18]	2019	America	Dried skulls	Yes (other)	1000	468	254	214	Bilateral	Small	High	No			
			Imaging	No	170	77	32	45	Unilateral	Large	High	No			
Maletin et al. [20]	2020	Europe	Dried skulls	No	26	16	14	2	Bilateral	Small	Moderate	No			
Martinez et al. [21]	2014	America	Dried skulls	No	53	18	6	12	Unilateral	Small	Moderate	No			
Murlimanju et al. [23]	2015	Asia	Dried skulls	Yes (other)	78	29	13	16	Unilateral	Small	High	No			
Natsis et al. [24]	2018	Europe	Dried skulls	Yes (wire)	195	78	42	36	Bilateral	Large	High	Yes (IAS)	2.71	2.31	
Nayak et al. [25]	2018	Asia	Dried skulls	Yes (other)	30	9	6	3	Bilateral	Small	High	Caliper (yes)	1.26	1.80	
Ozer and Govsa [26]	2014	Asia	Dried skulls	No	172	60	16	44	Unilateral	Small	Moderate	Yes (IAS)	0.97	2.38	10.59
Raval et al. [28]	2015	Asia	Dried skulls	Yes (wire)	150	61	29	32	Unilateral	Small	Moderate	Yes (caliper)	1.05		
Reymond et al. [29]	2005	Europe	Dried skulls	Yes (wire)	100	17	5	12	Unilateral	Small	Moderate	Yes (IAS)			
Rossi et al. [30]	2010	America	Dried skulls	No	80	32	11	21	Unilateral	Small	Moderate	Yes (caliper)	1.52	2.16	
Sharma and Garud [31]	2011	Asia	Dried skulls	Yes (wire)	50	31	22	9	Bilateral	Small	Moderate	No			
Shinohara et al. [32]	2010	America	Dried skulls	Yes (wire)	400	135	62	73	Unilateral	Large	Moderate	Yes (IAS)	0.71	2.57	11.24
Toledo junior et al. [33]	2016	America	Dried skulls	Yes (other)	84	35	14	21	Unilateral	Small	Moderate	No			

*T* total, *B/L* bilateral, *U/L* unilateral, *DV* DICOM Viewer, *IAS* image analysis software

### Prevalence of the sphenoidal emissary foramen (SEF)

**Fig. 2:**
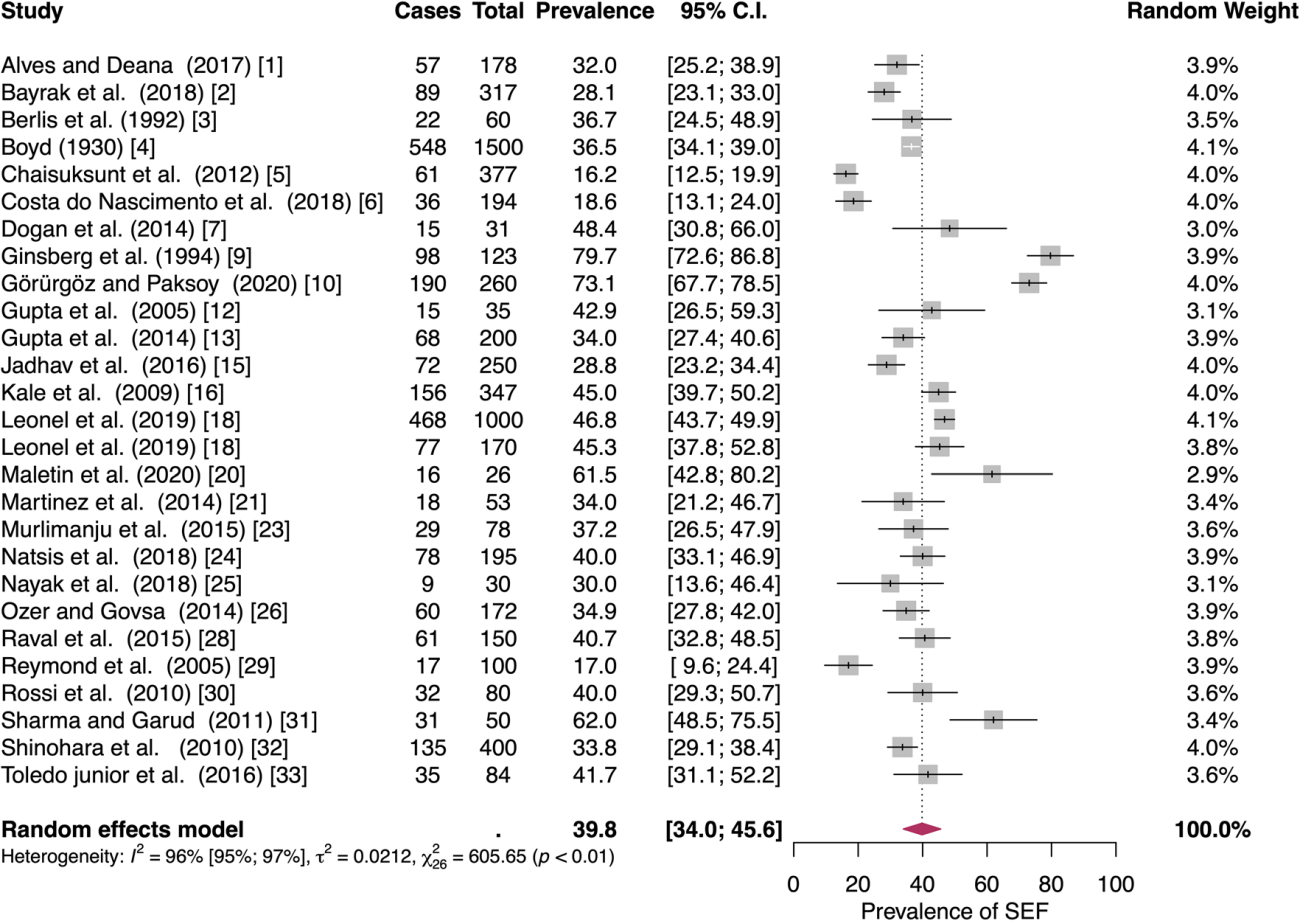
Forest plot evaluating the calculated prevalence of the sphenoidal emissary foramina (SEF) using random-effects model

**Fig. 3:**
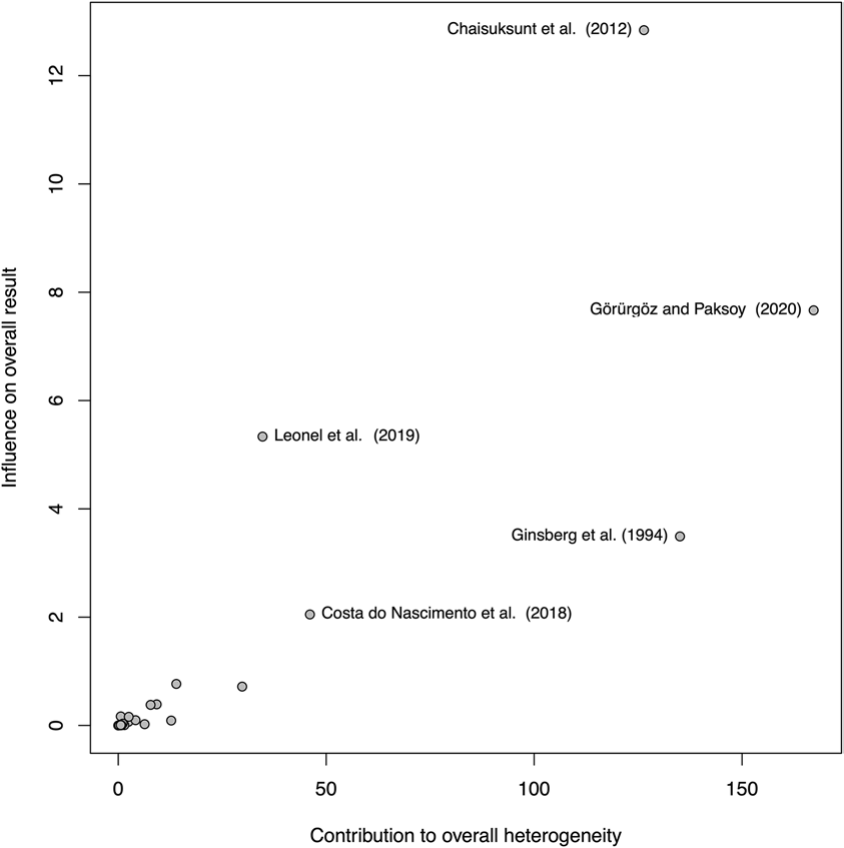
Diagnostic plot (Baujat plot) for the detection of heterogeneity sources in meta-analytic data. On the horizontal axis, the contribution of each study to the overall Q-test statistic is displayed

**Fig. 4:**
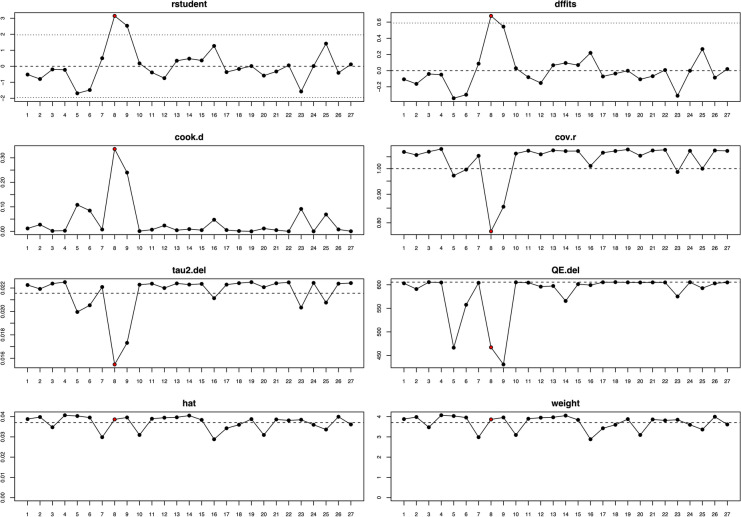
Visual representation of the influence diagnostics for each of the included studies. Influential studies are marked as red dots. Abbreviations used—rstudent: studentized deleted residuals; dffits: DFFITS values; cook.d: Cook’s distances; cov.r: covariance ratio; tau2.del: estimated τ^2^ values; QE.del: estimated Cochran’s *Q* values

**Fig. 5:**
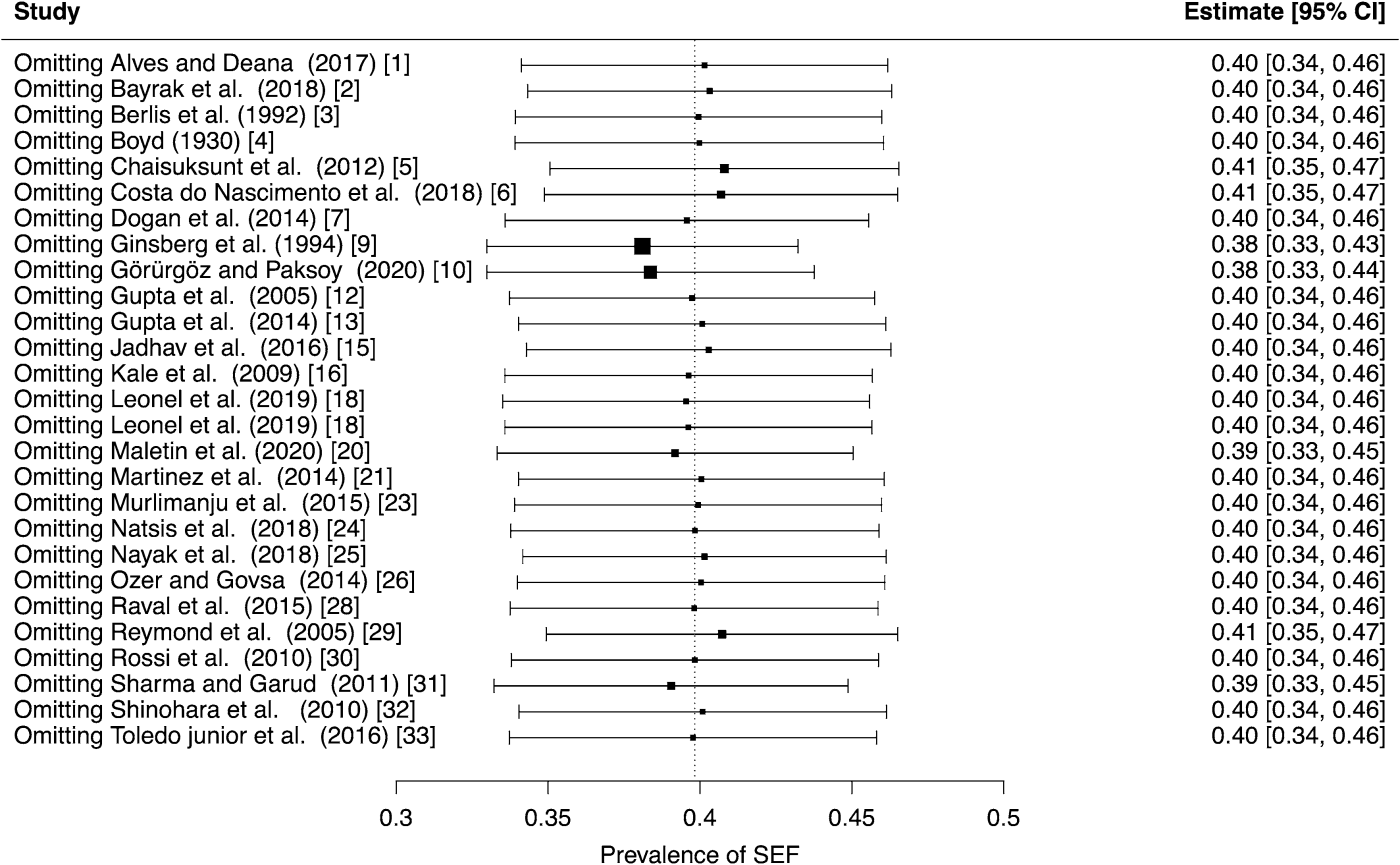
Forest plot displaying the re-calculated pooled effects, with one study omitted each time, using the leave-one-out method. The further a box deviates from the reference line, the more pronounced the impact of the corresponding missing study will be on the original summary proportion

A random-effects model analysis yielded an initial overall SEF prevalence of 39.8% (95% CI 34.0−45.7) (Fig. 2).The estimated heterogeneity was statistically significant (*p* < 0.001), and of high magnitude (*I*^2^ = 95.8%; 95% CI 92.9–97.7). The Baujat plot and the influence diagnostics are presented in Figs. 3 and 4. According to them, even though several studies were initially identified as outliers, the diagnostics indicated that only the Ginbserg et al. [10] study had an influential effect. The forest plot illustrating the results of the leave-one-out analyses is presented in Fig. 5. After the exclusion of the relevant study, the new pooled SEF prevalence was estimated at 38.1% (95% CI 32.8–43.4) with a reduction of 1.2% in the *I*^2^ being noticed (*I*^2^ = 94.6%; 95% CI 93.2–95.8).

### Publication bias and small-study effect

**Fig. 6:**
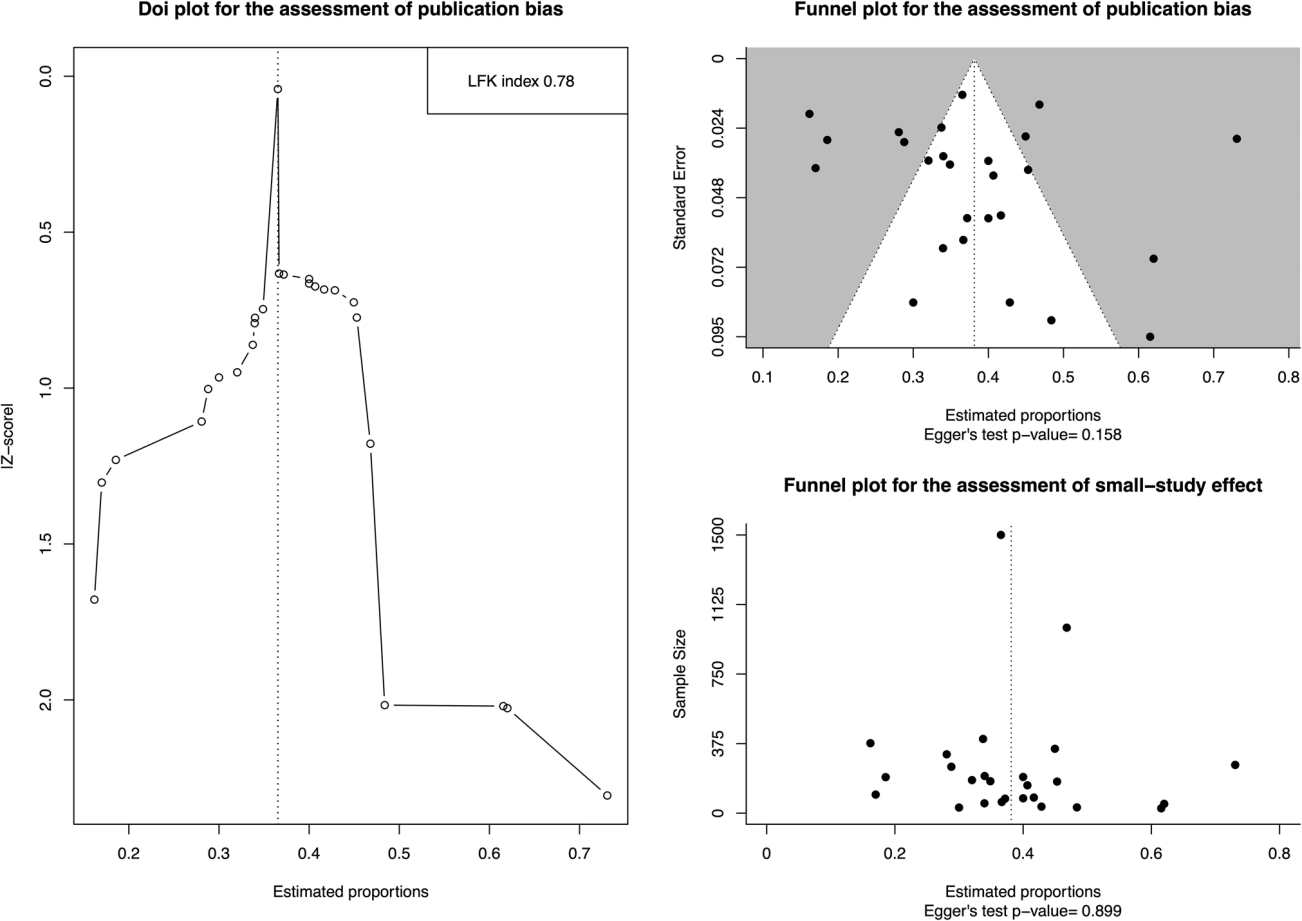
Depiction of the produced plots for the detection of publication bias (plots **a**, **b**) and small-study effect presence (plot **c**). The estimation of each plot’s asymmetry was performed by calculating the LFK index for plot (**a**) and Egger’s tests’ *p* value for plots (**b**) and (**c**)

Both the produced Doi and funnel plots (Fig. 6) for the assessment of presence of publication bias were assessed as asymmetric implying that bias might be present. However, the estimated LFK index (LFK index = 0.78) and the Egger’s test *p* value for the quantification of each plots’ asymmetry, respectively, were not deemed consistent with publication bias. As per the small-study effect, according to the data presented in Table 2 as well as on the interpretation of the produced funnel plot (Fig. 6) and the respective Egger’s test *p *value, the SEF prevalence was not moderated by the sample size. Therefore, no small-study effect was present.

**Table 2:** The results of the subgroup analysis on the differences of the subjects’ continent of origin, type of data, probing, sample size, laterality, and study quality on the estimated prevalence

Predictor	Moderator (subgroup)		k	Prevalence (95% CI)	Q_M_	Q_E_
Continent of origin	*Europe*		5	36.2% (26.7–45.8)	0.845	< 0.0001
	*America*		8	36.4% (28.4–44.4)		
	*Asia*		13	39.9% (29.7–50.1)		
Type of data	*Imaging*		3	48.8% (20.1–77.5)	0.401	< 0.0001
	*Dried skulls*		23	36.4% (31.7–41.0)		
Probing	*No*		11	35.8% (30.4–41.1)	0.508	< 0.0001
	*Yes*		15	39.2% (30.7–47.6)		
Instrument used	*Wire*		7	40.2% (23.3–57.0)	0.941	< 0.0001
	*Bristle*		3	32.5% (26.7–38.3)		
	*Other*		5	43.5% (39.3–47.7)		
Dominance	*Unilateral*		16	33.1% (28.1–38.1)	**0.016**	< 0.0001
	*Bilateral*		10	46.0% (36.8–55.3)		
Sample size	*Small*		17	37.5% (31.9–43.2)	0.842	< 0.0001
	*Large*		9	38.6% (29.3–48.0)		
Study quality	*Moderate*		17	35.7% (31.4–40.1)	0.473	< 0.0001
	*High*		9	40.4% (28.4–52.5)		
Measurements	*No*		12	41.5% (36.9–46.2)	0.198	< 0.0001
	*Yes*		14	34.8% (25.6–44.0)		
Instrument used	*Caliper*		6	34.0% (24.6—43.5)	0.084	< 0.0001
	*DICOM Viewer*		3	46.0% (13.3—78.8)		
	*Image analysis software*		5	28.3% (18.2—38.3)		

In bold text, the statistically significant findings are being noted

*k* number of studies combined, *Q*_*M*_* p value of the t*est of moderators, *Q*_*E*_* p value of the t*est of residual heterogeneity

### Moderator analysis

The results of the subgroup analyses are summarized in Table 2. The SEF prevalence varied significantly only by the type of dominance (*p* = 0.016). The results of the performed regression analyses display the existence of a statistically significant, and a marginally non-significant association of the reported SEF prevalence with dominance (*p* = 0.005), and type of data (*p* = 0.060), respectively (Table 3). Specifically, according to the multivariable regression results, the unilateral dominance was associated with a 13.0 percentage points decrease in the reported SEF prevalence. In other words, when a sample of dried skulls is examined, the reported SEF prevalence will be 13.0% smaller if the frequency of the identified unilateral foramina exceeds the one of the bilateral. This finding highlights the necessity of the simultaneous recording and reporting of the unilateral and bilateral SEF prevalence (laterality-specific prevalence). The performed moderator analyses explained 32.3% of the residual heterogeneity (*R*^*2*^ = 32.3%).

**Table 3:** The output of the univariable and multivariable linear meta-regression analyses performed for the association of the sphenoidal emissary foramen (SEF) presence with the studied variables

	Univariable models			Multivariable model		
	Estimate	*p *value	95% CI	Estimate	*p *value	95% CI
**Continent of origin**						
* America (ref.)*						
* Asia*	0.03	0.597	(− 0.09; 0.15)			
* Europe*	0.01	0.956	(− 0.15; 0.16)			
**Type of data**						
* Dried skulls (ref.)*						
* Imaging*	0.12	0.106	(− 0.03; 0.27)	0.13	0.060	(− 0.01; − 0.26)
**Probing**						
* No (ref.)*						
Yes	0.02	0.768	(− 0.09; 0.12)			
Instrument used						
* Bristle*	− 0.02	0.778	(− 0.21; 0.16)			
* Wire*	0.03	0.705	(− 0.11; 0.16)			
* Other*	0.02	0.705	(− 0.13; 0.18)			
**Dominance**						
* Bilateral (ref.)*						
* Unilateral*	− 0.13	0.010**	(− 0.22; − 0.03)	− 0.13	0.005**	(− 0.22; − 0.04)
**Study sample size**						
* Large (ref.)*						
* Small*	− 0.01	0.880	(− 0.12; 0.10)			
**Study Quality**						
* High (ref.)*						
* Moderate*	− 0.04	0.482	(0.32; 0.49)			
**Measurements**						
* No (ref.)*						
* Yes*	− 0.08	0.140	(− 0.18; 0.02)			
**SEF diameter**	0.02	0.787	(− 0.13; − 0.17)			
**SEF–FO distance**	− 0.06	0.527	(− 0.27; − 0.15)			
**SEF–FS distance**	− 0.35	0.194	(− 1.12; − 0.43)			

In bold text, the statistically significant findings are being highlighted

*Ref* reference category, *95% C.I. 95%* confidence intervals, *SEF diameter* sphenoidal emissary foramen’s anteroposterior diameter (measured in mm), *SEF–FO distance* distance between sphenoidal emissary foramen and foramen ovale (measured in mm), *SEF–FS distance* distance between sphenoidal emissary foramen and foramen spinosum, ** strong statistical association

### Study’s limitations

First, it should be noted that the unidentified heterogeneity remains on moderate levels. This indicates that the reported summary estimates must be interpreted with caution. Moreover, the small number of imaging studies and articles from various geographic locations (e.g., Europe), as well as the lack of a “gold standard” for measuring foramina dimensions and relative distances should be considered. Therefore, more effort should be made toward this direction.

## Conclusion

The SEF prevalence is estimated at 38.1%. The unilateral dominance is associated with a decrease in the SEF prevalence. Therefore, laterality-specific estimates should be established and followed for the estimation of the emissary foramina prevalence.

## Data Availability

Literature and Rstudio data are available if requested.

## Abbreviations

FO: Foramen ovale
FS: Foramen spinosum
FV: Foramen of Vesalius
SEF: Sphenoidal emissary foramen
SEV: Sphenoidal emissary vein
CI: Confidence intervals
SEF–FO: Sphenoidal emissary foramen to foramen ovale distance
SEF–FS: Sphenoidal emissary foramen to foramen spinosum distance
